# Outcomes of Endoscopic Intervention Using Over-the-Scope Clips for Anastomotic Leakage Involving Secondary Fistula after Gastrointestinal Surgery: A Japanese Multicenter Case Series

**DOI:** 10.3390/diagnostics13182997

**Published:** 2023-09-19

**Authors:** Naoya Tada, Hideki Kobara, Tomoaki Tashima, Hayato Fukui, Satoshi Asai, Takumi Ichinona, Koji Kojima, Kunihisa Uchita, Noriko Nishiyama, Joji Tani, Asahiro Morishita, Akihiro Kondo, Keiichi Okano, Hajime Isomoto, Kazuki Sumiyama, Tsutomu Masaki, Osamu Dohi

**Affiliations:** 1 Department of Gastroenterology and Neurology, Faculty of Medicine, Kagawa University, Takamatsu 761-0793, Japan; kobara.hideki@kagawa-u.ac.jp (H.K.); nishiyama.noriko@kagawa-u.ac.jp (N.N.); tani.joji@kagawa-u.ac.jp (J.T.); morishita.asahiro@kagawa-u.ac.jp (A.M.); masaki.tsutomu@kagawa-u.ac.jp (T.M.); 2 Department of Endoscopy, The Jikei University School of Medicine, Tokyo 105-8461, Japan; kazusumi735@gmail.com; 3 Department of Gastroenterology, Saitama Medical University International Medical Center, Saitama 350-1298, Japan; t.tashima1981@gmail.com; 4 Molecular Gastroenterology and Hepatology, Graduate School of Medical Science, Kyoto Prefectural University of Medicine, Kyoto 602-8566, Japan; h-fukui@koto.kpu-m.ac.jp (H.F.); osamu-d@koto.kpu-m.ac.jp (O.D.); 5 Department of Gastroenterology, Tane General Hospital, Osaka 550-0025, Japan; bonyaritetsu1226@hotmail.co.jp (S.A.); icnntakumi@yahoo.co.jp (T.I.); 6 Department of Gastroenterology, Kochi Red Cross Hospital, Kochi 780-0026, Japan; kozikozima22@gmail.com (K.K.); ucchy31@yahoo.co.jp (K.U.); 7 Department of Gastroenterological Surgery, Faculty of Medicine, Kagawa University, Takamatsu 761-0793, Japan; kondo.akihiro.z4@kagawa-u.ac.jp (A.K.); okano.keiichi@kagawa-u.ac.jp (K.O.); 8 Division of Gastroenterology and Nephrology, Faculty of Medicine, Tottori University, Tottori 683-8504, Japan; isomoto@tottori-u.ac.jp

**Keywords:** over-the-scope clip, anastomotic leakage, fistula, endoscopic closure

## Abstract

Background: The over-the-scope clip (OTSC) is a highly effective clipping device for refractory gastrointestinal disease. However, Japanese data from multicenter studies for anastomotic leakage (AL) involving a secondary fistula after gastrointestinal surgery are lacking. Therefore, this study evaluated the efficacy and safety of OTSC placement in Japanese patients with such conditions. Methods: We retrospectively collected data from 28 consecutive patients from five institutions who underwent OTSC-mediated closure for AL between July 2017 and July 2020. Results: The AL and fistula were located in the esophagus (3.6%, *n* = 1), stomach (10.7%, *n* = 3), small intestine (7.1%, *n* = 2), colon (25.0%, *n* = 7), and rectum (53.6%, *n* = 15). The technical success, clinical success, and complication rates were 92.9% (26/28), 71.4% (20/28), and 0% (0/28), respectively. An age of <65 years (85.7%), small intestinal AL (100%) and colonic AL (100%), defect size of <10 mm (82.4%), time to OTSC placement > 7 days (84.2%), and the use of simple suction (78.9%) and anchor forceps (80.0%) were associated with higher clinical success rates. Conclusion: OTSC placement is a useful therapeutic option for AL after gastrointestinal surgery.

## 1. Introduction

**Figure 1:**
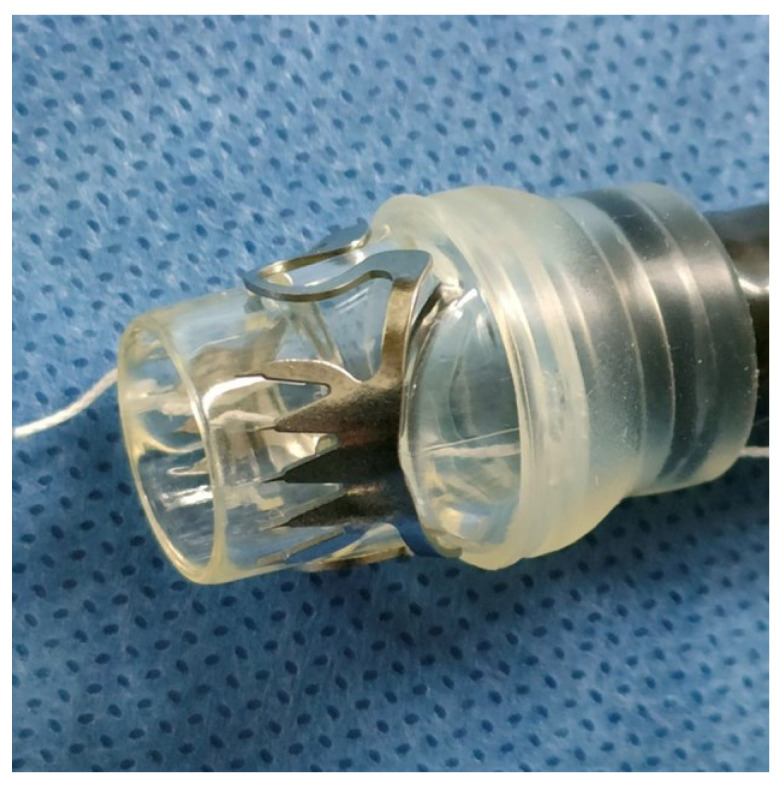
Over-the-scope clip attached to an endoscope.

Anastomotic leakage (AL) after gastrointestinal surgery is a major complication that causes increased mortality [1,2,3]. AL is defined as disruption at a surgical anastomosis resulting in fluid collection with or without evidence of the extravasation of contrast medium on radiologic evaluation. AL is one of the most serious complications encountered by surgeons, and it remains a challenge despite the development of medical technology. The incidence of AL is reportedly 11.4% after esophagectomy [4], 1.6% to 13.6% after gastrectomy for malignant tumors [5,6], and 3% to 33% after colorectal cancer surgery [7,8]. Occasionally, AL leads to the refractory condition of secondary fistula, which is defined as an abnormal communication between the epithelialized surface and peritoneal cavity. AL is mainly managed conservatively by nutritional support and infection control with antibiotics, but drainage is often required after a long duration of treatment [2,9]. Surgical interventions might be required when sepsis, a secondary fistula, and other refractory conditions occur [10,11,12,13]. However, several methods of endoscopic intervention have been reported, including tissue sealing using fibrin glue or cyanoacrylate [14,15,16], metal stents [17,18,19,20,21,22], endoscopic vacuum therapy [23,24,25], and suturing devices [26,27]. However, these techniques are often challenging because of a lack of certainty and evidence. Recent reports have described the efficacy of the over-the-scope clip (OTSC) system (Ovesco Endoscopy AG, Tübingen, Germany), a clipping device for the strong closure of tissue defects (Figure 1), for salvage treatment of refractory gastrointestinal diseases such as bleeding, perforation, AL, and fistulas [28,29,30]. However, few multicenter studies, especially Japanese studies [31], have systematically demonstrated the effectiveness of OTSC placement for AL and secondary fistulas by considering various factors presumed to affect the success of OTSC intervention [28,29]. Therefore, we conducted a multicenter study in Japan to investigate the characteristics of AL indicated for OTSC.

## 2. Materials and Methods

### 2.1. Study Design

**Figure 2:**
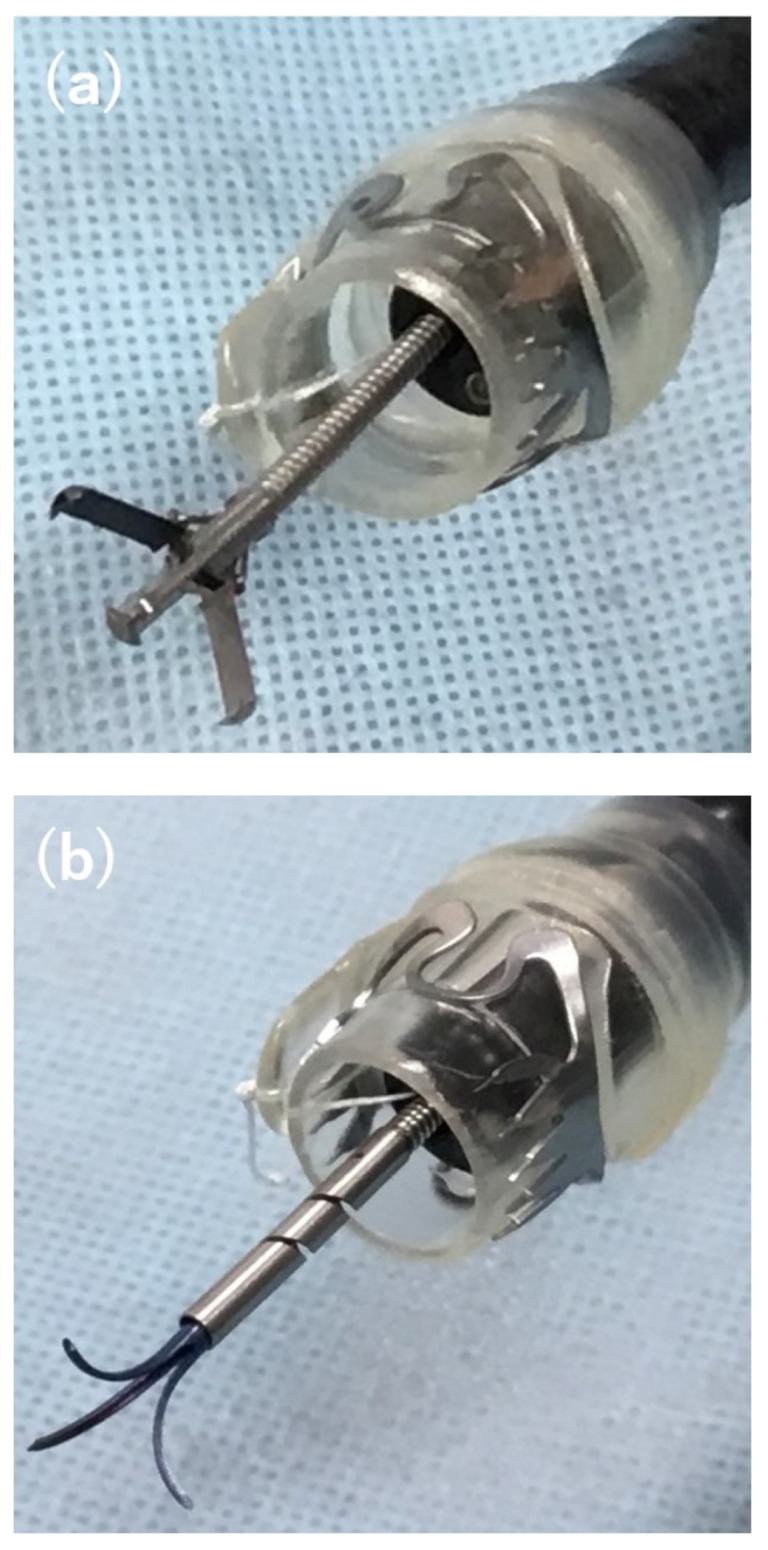
Two accessory devices. (**a**) Anchor forceps. (**b**) Twin Grasper.

This retrospective study was conducted at five institutions in Japan: Kagawa University Hospital, Saitama Medical University International Medical Center, Kyoto Prefectural University Hospital, Tane General Hospital, and Kochi Red Cross Hospital. All institutions had an Endoscopic Unit, and an OTSC was readily available. Patients who developed AL or a secondary fistula after gastrointestinal surgery and underwent endoscopic treatment with an OTSC were eligible for enrollment. Patients who did not provide written informed consent before OTSC placement were excluded. We enrolled 28 consecutive patients in whom OTSCs were used to treat AL or a secondary fistula after gastrointestinal surgery between July 2017 and July 2020. We collected data regarding patient characteristics, including sex, age, defect size, defect location, duration from the onset of AL or fistula to OTSC placement, suction method, and number of OTSCs used per patient. Diagnosis of AL and fistula was based on clinical symptoms such as fever and increased drainage, along with imaging examinations such as computed tomography, contrast examinations, and endoscopic examinations. A fistula was defined as an abnormal communication between two epithelialized surfaces. Postoperative AL represented one type of fistula, and was defined as the discontinuity of tissue apposition in the immediate postoperative period [32]. Suction was performed using simple suction, anchor forceps (Ovesco Endoscopy AG) (Figure 2a), or a Twin Grasper (Ovesco Endoscopy AG) (Figure 2b).

This study was approved by the Ethics Committee of Kagawa University (approval No. 2021-130, approval date: 10 February 2023) and was performed in accordance with the Declaration of Helsinki.

### 2.2. OTSC System

The OTSC is a strong clip with multiple sharp tips on either side attached to the applicator cap. Its mechanism and procedures are similar to those of endoscopic variceal ligation in that the target is suctioned into the cap and strangled by the device. A major advantage of the OTSC system is its powerful grip [33], leading to a higher overall clip retention rate [34]. The key to successful OTSC placement is to suction the target lesion to the application cap [31]. In most cases in this study, the simple suction method was first attempted. When simple suction was not suitable for suctioning the intestinal wall into the cap, an accessory device such as Anchor forceps or the Twin Grasper was used. Anchor forceps, which consist of three movable needles, is suitable for grasping hard intestinal wall components such as fistulas. By contrast, the Twin Grasper is suitable for soft or thin intestinal walls and large defects because the forceps on both sides grasp the edge of the defect [34]. Two types of OTSC were used in this study: gastrostomy closure (gc) and traumatic (t) types. The gc type has long, large claws and is mainly suitable for gastric lesions. The t type has short sharp claws and is suitable for small intestinal or colonic lesions with thin intestinal walls.

### 2.3. OTSC Procedures

The OTSC procedure is composed of several steps:

Step 1. An application-cap-mounted OTSC is attached to the endoscope considering the direction of the claws for each AL site.
Step 2. The endoscope is inserted into the gastrointestinal tract to the AL site.
Step 3. The target defect is suctioned into the application cap using simple suction, Anchor forceps, or the Twin Grasper.
Step 4. The OTSC is released by quicky rotating the handwheel attached to the forceps channel of the endoscope.
Step 5. After OTSC placement, endoscopic confirmation is performed to ensure that no defects remain. Fluoroscopic contrast or indigo carmine tests can be performed if necessary.

The indications for OTSC placement in each case, the number of OTSCs placed, the suction method, and the type of endoscope used for OTSC placement were determined at the discretion of the endoscopist in this study. The OTSC procedures were performed by endoscopists who had performed more than three procedures. All patients received conservative treatment with infection control before and after OTSC placement, including continuous drainage, empirical broad-spectrum antibiotics, and nutritional support.

### 2.4. Outcome Measures

The outcomes of interventions using OTSCs were the technical success, clinical success, and complication rates. Technical success was defined as macroscopic disappearance of the defect macroscopically following OTSC placement regardless of whether a gastrointestinal contrast examination was performed. Clinical success was defined as resolution of the AL and fistula (i.e., disappearance of digestive fluid leakage, complete closure of the defect, and absence of fever and abscess) within 30 days after OTSC placement without the use of other interventions. Complications were defined as any medical problems that occurred during or after the procedure, including gastrointestinal perforation or bleeding caused by injury from the OTSC claws or intraluminal stenosis after the OTSC closure. Short-term outcomes were evaluated within the first 30 days after OTSC placement. Continuous variables were presented as the mean ± SD or median (range).

## 3. Results

The patients’ median age was 75 years (range, 42–85 years). The median duration from diagnosis of AL and fistula to OTSC placement was 21 days (range, 0–668 days). The mean defect size was 7.6 ± 3.7 mm. The mean defect size among patients with a duration of ≤7 and >7 days between diagnosis and OTSC placement was 7.8 ± 3.9 and 7.5 ± 4.0 mm, respectively. The AL and fistula sites included the esophagus (3.6%, *n* = 1), stomach (10.7%, *n* = 3), small intestine (7.1%, *n* = 2), colon (25.0%, *n* = 7), and rectum (53.6%, *n* = 15). The indications for surgery were malignant tumors in 25 (89.2%) patients, followed by bleeding, perforation, and diverticulitis of the colon in 1 (3.6%) patient each. AL and secondary fistulas were present in 14 (50.0%) patients each. All surgeries involved gastrointestinal resection. The suction methods of OTSC were simple suction (67.9%, *n* = 19, anchor forceps (17.9%, *n* = 5) and the Twin Grasper (14.2%, *n* = 4). The mean number of OTSCs used per patient was 1.4 ± 0.4. The patients’ characteristics are presented in Table 1 and Table 2.

**Figure 3:**
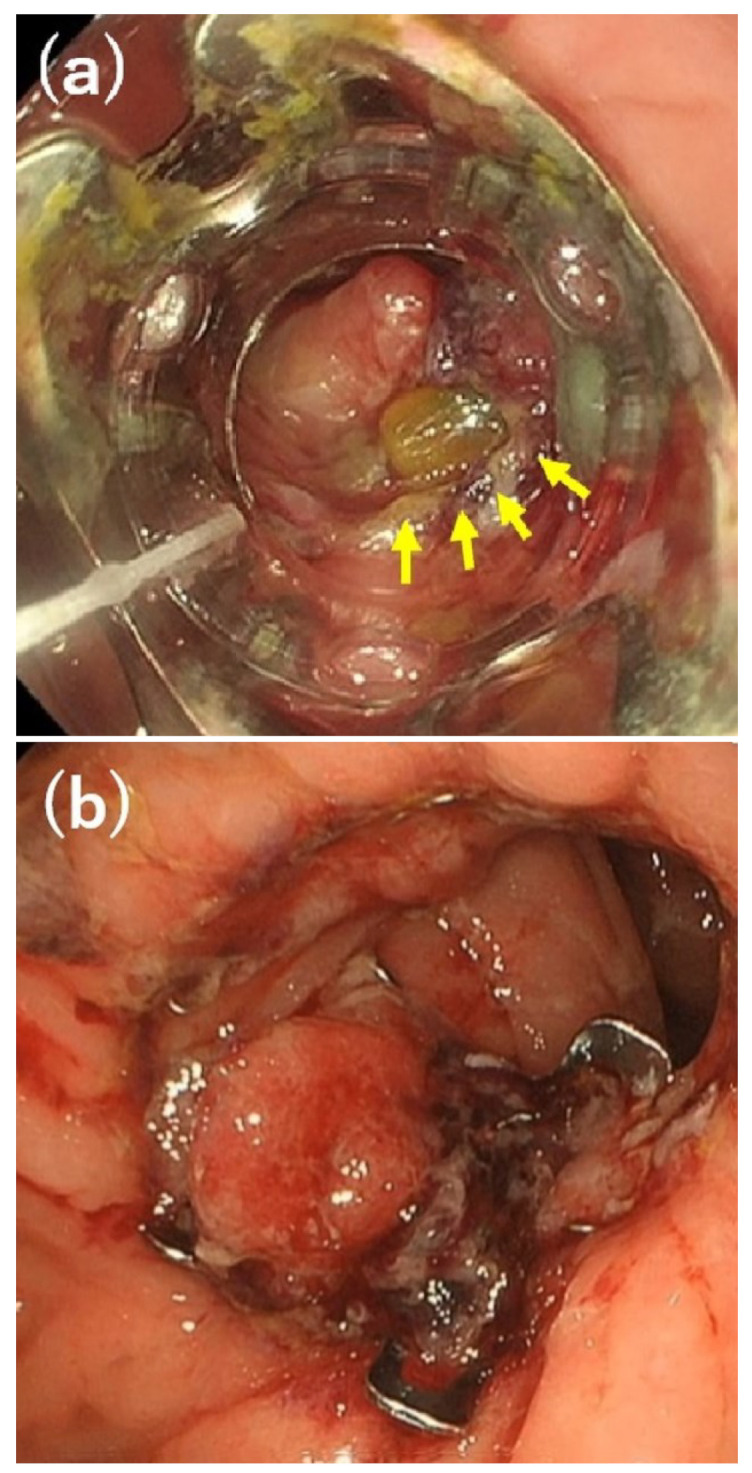
A clinically successful case after bypass surgery of the small intestine and colon. (**a**) A secondary fistula of 5 mm in size (yellow arrows) with a tip of the yellow drain on day 34 after the diagnosis of anastomotic leak. (**b**) The defect was successfully closed using over-the-scope clip placement, and clinical success was achieved.

**Figure 4:**
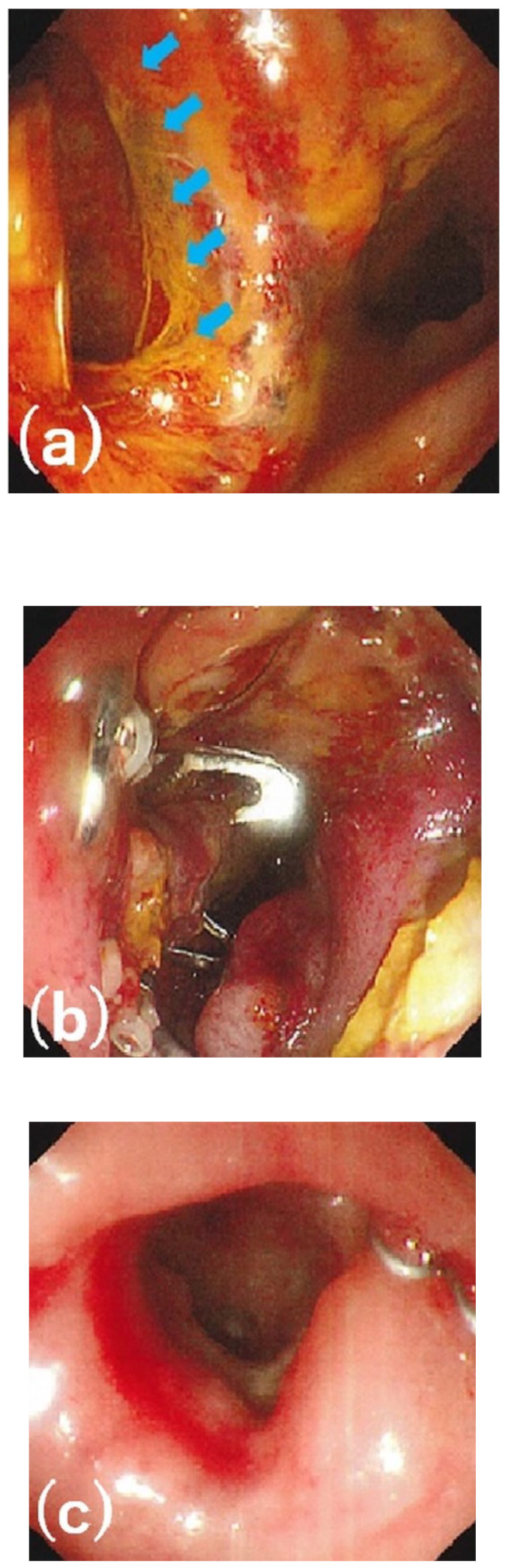
A clinically unsuccessful case of over-the-scope clip (OTSC) intervention after low anterior resection. (**a**) Anastomotic leakage (blue arrows) with severe inflammation on postoperative day 7. (**b**) Successful OTSC placement. (**c**) The OTSC fell off and the defect remained after 5 weeks.

The technical success, clinical success, and complication rates in all 28 patients were 92.9% (26/28 patients), 71.4% (20/28 patients), and 0.0% (0/28 patients), respectively, as summarized in Table 3. The rate of using other interventions after OTSC placement in clinically unsuccessful cases was 37.5% (3/8 patients). The clinical success rate by age (<65 vs. ≥65 years), location, defect size (<10 vs. ≥10 mm), duration from the diagnosis of AL to OTSC placement (≤7 vs. >7 days), and suction method is presented in Table 4. The clinical success rate was higher for patients aged <65 years (85.7%), those with small intestinal (100%) or colonic AL and fistulas (100%), those with defect sizes of <10 mm (82.4%), those with a >7-day duration between diagnosis and OTSC placement (81.2%), and those who underwent suction via simple suction (78.9%) and Anchor forceps (80.0%). The clinical success rates of AL and fistulas were 64.3% (9/14) and 78.6% (11/14), respectively. Among eight patients with OTSC failure, five continued conservative treatment, including antibiotics, drainage, and nutritional support after the OTSC intervention. Resolution of the condition took one to two months for four of these patients, whereas the remaining patient died of pneumonia during AL treatment. The surgical treatment of AL was performed in two patients. They underwent surgical repair of the leakage and colostomy with appropriate drainage, and both achieved improvement. One patient underwent endoscopic treatment with polyglycolic acid sheets and fibrin glue, but did not achieve improvement. The patient had an abscess with fever and continued conservative treatment as described above. The disappearance of the abscess and closure of the defect took approximately 2 months. Figure 3 and Figure 4 present a clinically successful case of OTSC placement and a clinically unsuccessful case of OTSC placement, respectively.

## 4. Discussion

AL is commonly caused by a postoperative anastomotic defect after surgery [32], and it is associated with high risks of morbidity and mortality. Antibiotics therapy and drainage are essential infection control measures in patients with AL [2,9]. The disappearance of the defect orifice is a key element to avoid the leakage of luminal contents and ensure the healing of the AL. Defect closure often requires substantial time to achieve when relying on conservative treatment with infection control, potentially leading to worse overall health and a poor nutritional status. Therefore, endoscopic interventions, including minimally invasive procedures, have been considered to accelerate healing. OTSC placement is a closure option for defects that are difficult to manage by endoscopy and that require surgical interventions [28,29]. However, few multicenter studies in Japan have demonstrated the outcomes of OTSC placement in patients with AL and a secondary fistula after gastrointestinal surgery. Moreover, the suitability of OTSC placement in patients with AL remains unclear. Therefore, we conducted this retrospective multicenter study in Japan to evaluate the efficacy and safety of OTSC placement for AL and secondary fistulas.

The clinical success rate was higher than that reported in a previous review (71.4% vs. 66.0%) [28]. Most defects could be successfully closed via OTSC placement, as indicated by the high technical success rate of 92.9%. Conversely, the clinical success rate was only 71.4%. The key to successful OTSC placement is adequate suctioning of the intestinal wall surrounding the defect into the cap [31]. Insufficient suction of the intestinal wall into the cap can lead to early OTSC loss. Therefore, the clinical success rate may be lower in conditions involving AL or fistulas because fibrosis of the intestinal wall is indurated around the defect in such cases. In a previous review, the clinical success rate for AL was 66.0% [28], which was lower than the clinical success rate of perforation (84.6%), implying difficulty of OTSC placement in fibrotic areas. Thus, the inability of the Twin Grasper to adequately grasp the intestinal wall in with the presence of fibrosis might be reflected by its lower clinical success rate of 25% (1/4 cases) in the present study. Conversely, the clinical success rate was higher in patients with small intestinal or colonic AL, but not in those with esophageal AL. Although the esophageal wall is commonly considered to be thin, it might not be suitable for OTSC procedures because of the narrow lumen, which limits maneuverability [31]. We presume that this is one reason for the lower number of patients with esophageal AL in the present study. In addition, the defect size is a relevant factor for the success of OTSC placement. Kobara et al. [28] concluded that the most suitable defect size for the placement of a single OTSC is <10 mm. Haito-Chavez et al. [35] reported a higher clinical success rate of 86.6% (26/30 cases) for the OTSC system in patients with AL after gastrointestinal surgery and a mean defect size of 8 mm (range, 5–10 mm). The present study also showed that the clinical success rate for patients with defect sizes of <10 mm was higher than for patients with larger defects. There were three cases of failure in patients with defect sizes of <10 mm, including one patient with esophageal AL and two patients with rectal AL. This finding might be attributable to insufficient suctioning of the intestinal wall because of limited maneuverability, as previously mentioned in the esophagus, and the thickness of the tissue in the rectum. The present study also showed higher efficacy in patients with a >7-day duration between diagnosis and treatment. In our opinion, most cases of AL, excluding those requiring early surgical intervention, can be treated conservatively in Japan using infection control and drainage at the beginning of the diagnosis, and cases that do not improve within 1–2 weeks are often considered refractory conditions that require other treatments such as endoscopic or surgical intervention. This could be one reason why many patients underwent OTSC placement more than 7 days after diagnosis in the study. In addition, in the early stages of AL, it might be difficult to achieve wound repair because of inflammation or ischemia even if the defect orifice is closed via OTSC placement.

The complications associated with the OTSC system should also be discussed. Several complications of OTSC placement have been reported, including injury of the defect orifice by Anchor forceps [36], esophageal perforation during insertion of the endoscope carrying the OTSC [37], microperforation of a duodenal ulcer [38] caused by the OTSC claws, and further enlargement of the perforation site [39]. A guidewire-assisted OTSC delivery method for prevention of injury of the intestinal wall while transporting the OTSC to the AL site has been described, and it appears useful for distal intestine lesions [40]. Gastrointestinal stenosis is the most common complication after OTSC placement [36,40]. Baron et al. [41] reported occlusion of the jejunum caused by multiple OTSC placements for iatrogenic perforation during endoscopic retrograde cholangiopancreatography using double-balloon endoscopy, leading to surgical intervention. Pham et al. [42] described one solution to this problem. Total occlusion of the esophagus occurred following OTSC placement for a fistula caused by sleeve gastrectomy, and they successfully removed the OTSC using a DC Clip Cutter (Ovesco Endoscopy AG). Although there are several reports of OTSC-associated complications, a review article identified a severe complication rate of only 0.59% (9/1517 cases) [28]. In addition, no OTSC-associated complications occurred in the present study. Although we must be vigilant regarding complications, the OTSC system can be considered a safe device.

The OTSC is a promising alternative option for closing AL sites because of its simplicity, rapid effect, and high efficacy. However, AL remains one of the most challenging targets for endoscopic gastrointestinal defect closure. OTSC application alone sometimes results in unsuccessful AL closure, thus necessitating combination strategies. In our opinion, when an indurated AL site cannot be closed by OTSC placement alone, combined use of tissue sealants such as polyglycolic acid sheets with fibrin glue or cyanoacrylate can be considered [16,43]. In particular, because cyanoacrylate solidifies quickly and acts fast, its antibacterial activity might be advantageous for AL. Validation of the efficacy of this combination method requires further investigation.

This study had several limitations. First, because this was a retrospective study, selection bias was unavoidable. Second, this study included a small number of patients with heterogeneous anastomoses. Therefore, it is difficult to determine the optimal patient cohort and timing for OTSC intervention. Third, when analyzing long-term outcomes, most patients could not be surveyed because they were transferred from our institutions to other hospitals. Therefore, we aim to conduct a prospective study to evaluate long-term outcomes. However, this study demonstrated that OTSC intervention is useful and effective for AL and secondary fistulas by assessing current real-world clinical outcomes. Furthermore, we believe that the present data will contribute to the establishment of criteria and an algorithm for OTSC intervention to treat AL in future prospective studies. A randomized controlled trial is required to evaluate the optimal use of OTSCs for AL and secondary fistulas.

## 5. Conclusions

This study provided real-world data for OTSC placement as a salvage therapy for AL in Japanese patients. OTSC placement is effective and safe as a minimally invasive treatment for AL after gastrointestinal surgery.

## Figures and Tables

**Table 1:** Summary of patient characteristics.

Sex, Male/Female, *n*	19/9
Age, year, median (range)	75 (42–85)
Duration from diagnosis to OTSC, day, median (range)	21 (0–668)
Defect size, mm, mean (±SD)	7.6 (±3.7)
Anastomotic leakage, n (%)	14 (50)
Secondary fistula, n (%)	14 (50)
≤7 days from diagnosis of AL to OTSC, mm, mean (±SD)	7.8 (±3.9)
>7 days from diagnosis of AL to OTSC, mm, mean (±SD)	7.5 (±4.0)
Location, n (%)	
Esophagus	1 (3.6)
Stomach	3 (10.7)
Small intestine	2 (7.1)
Colon	7 (25.0)
Rectum	15 (53.6)
Indication for surgery, n (%)	
Malignant tumor	25 (89.2)
Gastrointestinal bleeding	1 (3.6)
Gastrointestinal perforation	1 (3.6)
Diverticulitis	1 (3.6)
Suction method of OTSC, n (%)	
Simple suction	19 (67.9)
Anchor	5 (17.9)
Twin Grasper	4 (14. 2)
The type of OTSC (gc type/t type), n	3/25
The size of OTSC (9 mm/10 mm/11 mm), n	16/11/1
The number of OTSC used per patient, n, mean (±SD)	1.4 (0.4)

OTSC, over-the-scope clip; AL, anastomotic leakage; gc type, gastrostomy closure type; t, traumatic type; SD, standard deviation.

**Table 2:** Characteristics and outcomes of 28 patients with AL treated by OTSC.

Case	Age	Sex	Location	Indication for Surgery	Duration * (Day)	Defect Size(mm)	Type of Defect	Over-the-Scope Clip				Success		Complication	Other Intervention **
								Suction Method	Type	Size (mm)	Number	Technical	Clinical		
1	85	F	Rectum	Cancer	0	15	Leakage	Twin Grasper	t	9	2	Yes	No	No	Conservative
2	37	M	Colon	Perforation	0	10	Leakage	Simple suction	t	9	1	Yes	Yes	No	Conservative
3	79	F	Colon	Diverticulitis	31	5	Fistula	Anchor	t	9	1	Yes	Yes	No	Conservative
4	65	F	Colon	Diverticular bleeding	29	8	Fistula	Simple suction	t	9	2	Yes	Yes	No	Conservative
5	77	F	Colon	Cancer	60	8	Fistula	Simple suction	gc	12	1	Yes	Yes	No	Conservative
6	70	M	Stomach	Cancer	0	10	Leakage	Twin Grasper	gc	10	2	No	No	No	Fibrin glue with PGA
7	50	M	Colon	Cancer	34	15	Fistula	Simple suction	t	9	2	Yes	Yes	No	Conservative
8	50	M	Colon	Cancer	29	5	Fistula	Simple suction	gc	10	2	Yes	Yes	No	Conservative
9	80	M	Rectum	Cancer	28	10	Fistula	Simple suction	t	9	2	Yes	Yes	No	Conservative
10	73	M	Rectum	Cancer	199	10	Fistula	Simple suction	t	9	2	Yes	No	No	Conservative
11	79	F	Rectum	Cancer	283	10	Fistula	Simple suction	t	9	1	Yes	Yes	No	Conservative
12	54	F	Rectum	Cancer	285	3	Fistula	Simple suction	t	9	1	Yes	No	No	Conservative
13	73	M	Rectum	Cancer	4	5	Leakage	Simple suction	t	9	1	Yes	Yes	No	Conservative
14	66	F	Rectum	Cancer	21	2	Leakage	Simple suction	t	9	1	Yes	Yes	No	Conservative
15	77	M	Colon	Cancer	0	5	Leakage	Simple suction	t	9	1	Yes	Yes	No	Conservative
16	79	M	Rectum	Cancer	1	5	Leakage	Simple suction	t	10	2	Yes	Yes	No	Conservative
17	80	M	Rectum	Cancer	21	10	Fistula	Simple suction	t	9	2	Yes	No	No	Conservative
18	74	M	Rectum	Cancer	3	8	Leakage	Simple suction	t	10	1	Yes	No	No	Surgery
19	61	M	Rectum	Cancer	668	5	Fistula	Simple suction	t	10	1	Yes	Yes	No	Conservative
20	72	M	Rectum	Cancer	0	10	Leakage	Twin Grasper	t	9	1	No	No	No	Surgery
21	79	M	Esophagus	Cancer	0	2	Leakage	Anchor	t	9	2	Yes	No	No	Conservative
22	42	F	Rectum	Cancer	113	7	Fistula	Anchor	t	10	1	Yes	Yes	No	Conservative
23	78	F	Stomach	Cancer	20	10	Leakage	Twingrasper	t	9	1	Yes	Yes	No	Conservative
24	82	M	Rectum	Cancer	133	2	Fistula	Anchor	t	10	1	Yes	Yes	No	Conservative
25	75	M	Small intestine	Sarcoma	8	5	Leakage	Anchor	t	10	1	Yes	Yes	No	Conservative
26	80	M	Small intestine	Cancer	8	15	Leakage	Simple suction	t	10	2	Yes	Yes	No	Conservative
27	79	M	Stomach	Cancer	10	7	Leakage	Simple suction	t	10	1	Yes	Yes	No	Conservative
28	48	M	Rectum	Cancer	23	6	Fistula	Simple suction	t	10	1	Yes	Yes	No	Conservative

AL, anastomotic leakage; OTSC, over-the-scope clip; M, male; F, female; PGA, polyglycolic acid sheets. * Duration from diagnosis to OTSC placement, ** Use of other intervention after OTSC placement.

**Table 3:** Summary of outcomes of OTSC placement for AL and secondary fistulas.

Technical Success, % (n/N)	92.9 (26/28)
Clinical success, % (n/N)	71.4 (20/28)
Complication, % (n/N)	0 (0/28)
Use of other intervention after OTSC in clinically unsuccess cases, % (n/N)	37.5 (3/8)
Surgical intervention, n	2
Fibrin glue with polyglycolic acid sheets, n	1

OTSC, over-the-scope clip; AL, anastomotic leakage.

**Table 4:** Clinical success rate according to different variables.

Variable	% (n/N)
Age	
<65 years	85.7 (6/7)
≥65 years	66.7 (14/21)
Location	
Esophagus	0 (0/1)
Stomach	66.7 (2/3)
Small intestine	100 (2/2)
Colon	100 (7/7)
Rectum	60 (9/15)
Anastomotic leakage	64.3% (9/14)
Secondary fistula	78.6% (11/14)
Defect size	
<10 mm	82.4 (14/17)
≥10 mm	54.5 (6/11)
Duration from diagnosis to OTSC	
≤7 days	44.4 (4/9)
>7 days	84.2 (16/19)
Suction method	
Simple suction	78.9 (15/19)
Anchor	80 (4/5)
Twin Grasper	25 (1/4)

OTSC; over-the-scope clip.

## Data Availability

Data are not publicly available because of the need to ensure the protection of personal data and medical confidentiality.
